# Theatre-Neuroscience: a brief perspective on the use of neuroscientific techniques to measure interpersonal coordination and the sense of self in theatrical contexts

**DOI:** 10.1093/oons/kvag012

**Published:** 2026-09-10

**Authors:** Dwaynica A Greaves

**Affiliations:** Social Neuroscience Lab, UCL Institute of Cognitive Neuroscience, Alexandra House, 17-19 Queen Square, London, WC1N 3AZ, United Kingdom

**Keywords:** Theatre-Neuroscience, Social Cognition, Interpersonal Coordination, Sense of self, Neuroaesthetics

## Abstract

Cognitive neuroscientists have collaborated with the performing arts industry by bringing multimodal techniques, i.e. physiological, behavioural, and neural measurements, to the investigation of social cognition within these artistic contexts. This brief perspective presents, discusses, and evaluates the emerging field of *‘Theatre-Neuroscience’*, which is an example of such collaboration. Here, I discuss the Theatre-Neuroscience research conducted to date, which has predominantly investigated the social-cognitive concepts of actors’ and audiences’ interpersonal coordination during live theatre performances, and actors’ sense of self whilst embodying a character, in both laboratory and live environments. Therefore, this brief perspective aims to present empirical support for the field’s value to cognitive neuroscience and the acting industry, exemplifying why interdisciplinary perspectives and paradigms are becoming increasingly favourable within cognitive neuroscience.

## Introduction

Theatre-Neuroscience is an emerging interdisciplinary field of research that utilises neuroscientific techniques to investigate the creation and experience of dramatic acting, and the effects of acting interventions on cognition and behaviour (as presented in Greaves et al. 2022). The integration of cognitive neuroscience and artistic disciplines has become increasingly desirable, with fields such as Neurocinematics and Neuroarchitecture also creating bridges between disciplines to further our understanding of social cognition, health and wellbeing (Hasson et al. 2008, Wang et al. 2022). The ideas underpinning Theatre-Neuroscience predate the use of a defined term, as scholars have explored the relationship between actors and audience members, and the actor and their character, before the publication of Theatre-Neuroscience experiments (Falletti et al. 2016, Kemp 2012, Mirodan 2015). The established fields of Empirical Aesthetics and Neuroaesthetics have paved the way for Theatre-Neuroscience to join the intersection of art, creativity, aesthetic experiences and empiricism (Fechner 1876, Semir Zeki 1999). Fechner (1876), the founder of Empirical Aesthetics, presented the idea of a bottom-up approach to investigating art, where an artwork is deconstructed into its parts for empirical exploration, furthering knowledge about human cognition and behaviour. Theatre-Neuroscience adopts Fechner’s bottom-up approach to its deconstruction of acting, guiding investigations into the effects of the acting experience on audiences, non-actor participants, and actors.

However, very little is known about the effects of acting on human cognition and behaviour compared to the initial art forms that inspired Empirical Aesthetics and Neuroaesthetics (i.e. visual arts, Goldstein 2009). Also, research into neural and behavioural responses to performing art forms such as dance has preceded theatre, providing examples of how cognitive neuroscientists can integrate performing arts into lab-based stimuli or integrate the lab into performing arts environments (Calvo-Merino et al. 2005, von Zimmermann et al. 2018). Nevertheless, Theatre-Neuroscience has strengthened the literature on measuring interpersonal coordination during live performances and has taken the lead in the investigations of the sense of self of performers (Brown et al. 2019, Greaves et al. 2022, Sun et al. 2023, Ward et al. 2018). Therefore, this brief perspective presents the field of Theatre-Neuroscience and discusses its preliminary yet novel investigations into the interpersonal coordination present between actors and audiences, and an actor’s sense of self. This discussion will lead to an evaluation of the research that has been conducted, with implications for future research at this intersection of disciplines.

## Interpersonal coordination during live theatre

Engaging in social interactions leads to a coordination of neural, physiological and behavioural signals, a phenomenon known as interpersonal coordination (Greaves et al. 2022, Schmidt and Richardson 2008). One form of interpersonal coordination is interpersonal synchrony, which refers to the implicit temporal coordination of actions between two individuals (Zamm et al. 2016). This concept is of great interest to social cognitive neuroscientists, as interpersonal synchrony has been found to predict pro-sociality in relation to group liking and cohesion (Cirelli 2018, von Zimmermann et al. 2018). The audience is an integral part of the acting industry, as the final production, whether on stage or film, is designed to communicate a message whilst providing an experience for the public (Karmakar 2013). Therefore, interdisciplinary research teams have developed an interest towards measuring whether interpersonal coordination is present during collective experiences of live theatre.

The first recorded investigation into the effects of watching a live theatre production on interpersonal coordination was conducted by researchers from University College London (UCL) and Lancaster University. This study aimed to serve the theatre industry rather than result in scientific publications, as reported in UCL News (UCL News 2017). Audience members watched a live performance of *Dreamgirls the Musical*, whilst their heart rate and electrodermal activity were measured with wrist-worn physiological sensors. Researchers found that audience members’ heart rates were in unison, speeding up and slowing down at the same rate during the performance. The significance of this finding is that these patterns, usually found in romantic couples (Coutinho et al. 2021), suggest that implicit social connections can be ignited while attending a theatre performance with strangers.

The first measurement of interpersonal synchrony in live adult theatre audiences was conducted by Sun et al. (2023). In this study, researchers measured interpersonal synchrony in actors and audience members during a live adaptation of Shakespeare’s *A Midsummer Night’s Dream*. The live performance included public engagement segments and an audience participatory period, where a few audience members were invited to perform on stage with the actors. Participants wore accelerometers within headbands and either remained seated through the performance or took part in the participatory period. Findings revealed that the participatory period resulted in increased interpersonal synchrony between actors and audience members who participated on stage, as well as between actors and a few seated audience members. Therefore, researchers concluded that increasing the proximity between actors and audience members (‘breaking the fourth wall’) can lead to increased implicit social connection. In addition, audience members who remained seated during the participatory period, yet had increased interpersonal synchrony with actors, may evidence activated action-observation/mirror neuron networks during live performance, as seen in lab-based dance-neuroscience research (Calvo-Merino et al. 2005). However, neural measurements would be necessary to strengthen these claims. Interpersonal synchrony was also found between actors as they performed across the two acts of the show, suggesting that, whether one is an actor or an audience member, engaging in a live theatre performance can ignite implicit social connection.

Applied theatre interventions in educational settings have been found, through behavioural tasks and self-report measures, to increase social-cognitive skills such as empathy, theory of mind and emotional regulation of children and adolescents compared to other creative interventions (Goldstein and Lerner 2018, Goldstein and Winner 2012). Therefore, measures of interpersonal synchrony in autistic children during a live theatre performance were conducted in collaboration with the theatre industry to challenge classifications of social-cognitive deficits across the autism spectrum (Leekam 2016, Volkmar and Reichow 2013). Ward et al. (2018) found interpersonal synchrony between a group of autistic children watching a *‘Hunter Heartbeat Method’* performance of Shakespeare’s *A Midsummer Night’s Dream* (Hunter 2014). Children were found to stim in time to the rhythm of the actors and instruments within the performance; furthermore, they exhibited eye contact and developed confidence to interact with actors during playful elements of the performance. Researchers concluded that their findings identify vital patterns of implicit coordination that can be easily missed by explicit observations, showing the importance of using neuroscientific techniques to investigate social-cognitive developments in modalities that may be missed by traditional observations, e.g. solely video coding. Therefore, the importance of researching the benefits of applied theatre is that it demonstrates how the intersection of theatre and neuroscience can be a form of empirical advocacy for vulnerable groups, in this case, neurotypical and neurodiverse children whose social-cognitive development has been found to benefit from funded intra- and extra-curricular theatre interventions (Goldstein and Winner 2024, Hunter 2014, Reading and Reading 2016).

Moving to the neural medium of coordination, brain–brain interpersonal coordination was found in the right frontopolar cortex (a region that contributes to self-other processing, Raposo et al. 2011) of pairs of actors rehearsing for a live performance of Shakespeare’s *A Midsummer Night’s Dream* (Greaves et al. 2022). Researchers further found that interpersonal synchrony was present between pairs of actors when rehearsing together, unrelated to the heart and breathing rate synchrony that naturally occurs when pairs of people are socialising (McFarland 2001, Wicher et al. 2026). Therefore, researchers concluded that there is something significant about actors performing together that results in neural and behavioural implicit social connection, providing empirical support for the pro-social benefits of acting professions.

## The actors’ sense of self

To create a holistic understanding of the social cognition that occurs in the context of acting, it’s important for Theatre-Neuroscience research to not only focus on the audiences of the art but also include the makers and performers of the art, investigating both ends of production. This direction of interest has predominantly focused on an actor’s sense of self. The sense of self is the awareness, processing and experience of a constant identity despite changes in one’s internal and external environment (Farmer and Tsakiris 2012, Mead 1913, Strohminger et al. 2017). One way that cognitive neuroscientists have operationalised the sense of self is to measure self-saliency during the presentation of self-other referential stimuli (Northoff et al. 2006). Therefore, cases where self-saliency may be compromised have presented themselves as insightful for these investigations. A case such as this is one of actors who, through acting techniques, have learned how to integrate the cognitions and physicality of self and character, theoretically compromising self-saliency for a realistic, organic performance (Berry and Brown 2019, Goldstein and Bloom 2011, Kemp 2012, Mirodan 2019).

**Figure 1:**
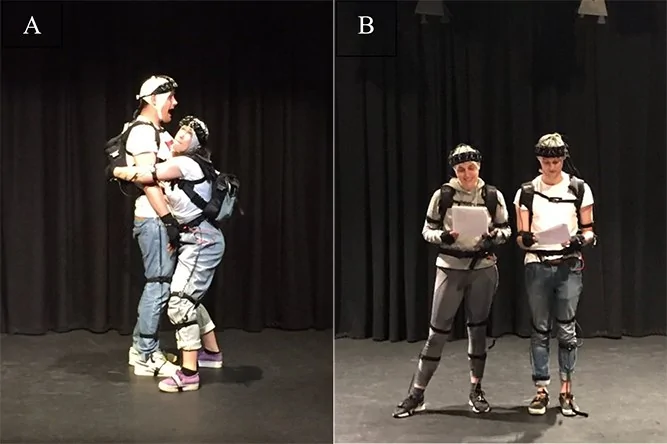
Images of *Deconstructing the Dream*’s hyperscanning experiment, which took place at UCL’s Bloomsbury Theatre drama studio (Greaves et al. 2022, images retrieved from https://deconstructingthedream.weebly.com/photos--video.html). A) Pairs of actors wearing functional near-infrared spectroscopy wearable systems (fNIRS, Shimadzu LIGHTNIRS, Japan) rehearsing a segment of Flute Theatre’s (London, UK) live adaptation of Shakespeare’s *A Midsummer Night’s Dream*. B) Pairs of actors completing the control task of reading a Shakespearean prologue out of character.

The first published empirical support for this theory was conducted by Brown et al. (2019). Here, an interdisciplinary team of researchers used functional magnetic resonance imaging (fMRI) to study the neural activity of university-trained method actors whilst in character. Actors were instructed to answer hypothetical questions, e.g. *‘Would you tell your parents you fell in love?’* as either themselves, a third-person close other, themselves in a British accent (as these were Canadian participants) or their character (Romeo or Juliet). Findings revealed that when answering questions as their character compared to themselves, actors had suppressed activation in neural regions associated with self-saliency (dorsomedial prefrontal cortex, superior frontal gyrus and ventral medial prefrontal cortex). Therefore, researchers concluded that being in character can lead to a suppression of self.

However, the constraint of using fMRI to study actors during performance is that their movement is severely restricted when lying still in an MRI scanner. This means that fMRI is not appropriate for research that requires actors to naturally use their physicality whilst in character. Therefore, Greaves et al. (2022) conducted a proof-of-principle study as part of the *‘Deconstructing the Dream’* project to test whether wearable functional near-infrared spectroscopy (fNIRS) could be used in a drama studio to investigate an actor’s sense of self and interpersonal coordination (Deconstructing the Dream 2019). Wearable fNIRS was instrumental in enabling the first real-world actor-actor hyperscanning (Hamilton 2021) experiment, as actors were able to navigate the rehearsal space in a natural way (Figure 1). Pairs of actors rehearsed for a live adaptation of Shakespeare’s *A Midsummer Night’s Dream* and completed control tasks whilst hearing the self-referential cue of their own and fellow actor’s name (adapting the name-call paradigm of Kampe et al. 2003). Findings revealed that actors had higher activation to hearing their own name in regions that contribute to self-other processing (left frontopolar and dorsolateral prefrontal cortex) during the control conditions rather than the acting condition. These findings align with Brown et al. (2019)’s conclusions of self-suppression whilst in character, providing further empirical support for the idea that self-saliency can be compromised by embodying a character.

Therefore, Brown et al. (2019) and Greaves et al. (2022) provide two different examples of how theatre and neuroscience have collaborated to fill gaps in the self-other processing literature, revealing cases where self-saliency can be compromised. While Brown et al. (2019) achieved this by demonstrating how performing arts can be integrated into the lab by creating Theatre-Neuroscience stimuli compatible with fMRI, Greaves et al. (2022) achieved this by demonstrating how the lab can be integrated into performing arts environments by using wearable neuroimaging with a drama studio.

## Evaluating Theatre-Neuroscience

Theatre-Neuroscience is an emerging field, as previously highlighted; therefore, the purpose of this brief perspective was to introduce the field, discuss its preliminary findings and now evaluate the research that has been conducted so far. Research on audiences during live performances has predominantly utilised physiological and behavioural measures to capture interpersonal coordination during live theatre. This has provided insight into the forms of implicit social connection between actors, between actors and audience members and between audience members (Sun et al. 2023, UCL News 2017). These insights evidence that pro-social behaviour is present in theatre spaces, whether one is an actor or audience member, which provides empirical support for the social-cognitive benefits of engaging with live theatre. This promotes the importance of attending live theatre despite rapid developments in streaming platforms and remote entertainment (Ginman 2013, Richardson 2015, Simonot-Bérenger et al. 2025). These findings have broader societal relevance, as interventions and activities combatting a lack of in-person social connectedness and increased loneliness have gained rapid importance (Nikolova 2023, Whitty and McLaughlin 2007), with health organisations turning to the arts for solutions, resulting in the emergence of interdisciplinary fields such as Creative Health (Gordon-Nesbitt 2017, Jenkins et al. 2025).

For neuroscience, the field demonstrates ways of measuring physiological and behavioural data from groups, expanding beyond pairs, within real-world social environments. This contributes to the growing literature that presents and examines the development of real-world neuroscientific paradigms with wearable technology (Hessels et al. 2020, Jones et al. 2025). Research into applied theatre has demonstrated how to appropriately and comfortably measure behavioural responses in neurodiverse populations, providing proof-of-principles for instances where neuroimaging systems are not appropriate (Ward et al. 2018). Furthermore, research has also revealed the social-cognitive benefits of theatre for autistic children, revealing that layers of social-cognitive interactions (e.g. eye contact, physical contact and rhythm) can be experienced by autistic children through theatrical interventions (Hunter 2014, Ward et al. 2018). The principal investigators of these research teams have continued to develop ‘human-centred’ research designs using these wearable sensors to measure interpersonal coordination in autistic children’s educational environments (Bell et al. 2022). Therefore, I argue that applied Theatre-Neuroscience research has begun to generate implications for education and policy, complementing existing research that demonstrates the benefits of funding theatre-based interventions for the development of social-cognitive skills, including mentalising, empathy, and emotional regulation, in children and adolescents (Goldstein and Winner 2012, 2024, Goldstein and Lerner 2018).

However, a gap remains in the audience research segment of the field, as there are currently no neuroimaging publications that have investigated live theatre audiences from a social-cognitive perspective. Therefore, questions about the neural effects of watching live theatre are yet to be answered. Despite this, interdisciplinary research teams have developed electroencephalography (EEG) neuroimaging studies testing novel multimodal developments in hyperscanning techniques, and mobile brain–body imaging during live theatre and dance performances (Hendry et al. 2025, Rai et al. 2025). These studies present the scope of live theatre research and contribute to the development of protocols and pipelines necessary for collecting and analysing the complex datasets that are derived from real-world environments.

Nevertheless, neuroimaging research into an actor’s sense of self has shed light on the neural networks active during an actor’s creative process, as well as revealing that concrete constructs such as self-saliency can be compromised by embodying a character (Brown et al. 2019, Greaves et al. 2022). This provides interesting grounds for cognitive neuroscientists to consider the abstractness of self-other interactions in the social world and how these interactions can affect the neural representation of the self. Further research into an actor’s sense of self is ongoing, with work yet to be published from my PhD thesis, international collaborations, and projects such as *‘The Role-Playing Brain’*, where researchers are currently replicating the work of Brown et al. (2019) with clinical implications for drama-based therapies (Berceanu et al. 2026). The development of these projects highlights the implications for the mental health of actors, providing a neuroscientific lens into the positive and negative effects of embodying a character and participating in public performance throughout one’s career (Carr and Lewis 2025, Cox 2024). Therefore, the field does not bias itself by investigating only the benefits of theatre for actors and audiences but also presents a contrasting perspective when investigating the effects of building and performing a character on an actor’s sense of self.

Overall, the audience-focused, applied, and actor-focused studies within Theatre-Neuroscience have begun to compile paradigms that either integrate the performing arts into the lab environment or integrate the lab into the real-world performance space, highlighting the flexibility of neuroscientific techniques. However, it is important to also highlight that the current Theatre-Neuroscience literature has predominantly focused on Western acting techniques and texts, as its publications span across the UK, Canada & USA (Brown et al. 2019, Falletti et al. 2016, Greaves et al. 2022, Kemp 2012). This is a reflection of the societies in which Theatre-Neuroscience ideas and research have developed. Therefore, as the field grows, the hope is to see Theatre-Neuroscience paradigms adapted across global approaches to acting, only where appropriate, as researchers must bear in mind that Western scientific methodologies may not be appropriate for investigating all acting techniques and practices. This argument is developed from knowledge that Western sciences have epistemological and ontological differences from Indigenous sciences (Stein et al. 2024), hence are not appropriate for the investigation of, for instance, Indigenous approaches to acting.

Lastly, I do not present a consensus of acceptance on the notion of interdisciplinary fields such as Theatre-Neuroscience. Valid concerns about the reductionism of the arts due to empirical approaches should continually be evaluated (Goede, 2014; Shimamura and Palmer, 2011). Also, further publications are needed to strengthen arguments for Theatre-Neuroscience’s impact on theatre, neuroscience and policy. Therefore, at this preliminary stage I argue that Theatre-Neuroscience best suits researchers and practitioners who seek an empirical approach to understanding the acting industry, its people, and its spaces, and to use these insights to contribute to the wider understanding of human cognition and behaviour.

## Conclusion

This brief perspective presented, discussed, and evaluated the emerging field of *‘Theatre-Neuroscience’*, with a focus on its investigations into the social-cognitive concepts of interpersonal coordination in actors and audiences and the sense of self of actors. The field’s research has shown preliminary implications for real-world neuroscientific methodologies, neurodiversity, mental health and wellbeing, exemplifying the value of interdisciplinary perspectives and paradigms within cognitive neuroscience. In conclusion, Theatre-Neuroscience is an example of what can be revealed about human cognition and behaviour when scientific and artistic disciplines integrate their knowledge and resources.

## Supplementary Material

Web_Material_kvag012

## Data Availability

No primary or secondary data were generated or analysed in support of this work.
