# Supplementary material for: Theatre-Neuroscience: a brief perspective on the use of neuroscientific techniques to measure interpersonal coordination and the sense of self in theatrical contexts
Source: Oxf Open Neurosci. 2026 Sep 10;5:kvag012. doi: 10.1093/oons/kvag012 (PMC13600366; doi:10.1093/oons/kvag012)
Supplement: Supplementary file 1 [file web_material_kvag012.docx]

**Decision Letter (OXFNSC-2026-001)**

**09-Feb-2026**

Oxford Open Neuroscience - Decision on Manuscript ID OXFNSC-2026-001

Dear Ms. Greaves,

Manuscript ID OXFNSC-2026-001 entitled "Theatre-Neuroscience: building interdisciplinary paradigms to investigate social cognition" which you submitted to the Oxford Open Neuroscience, has been reviewed. The comments of the reviewers are included at the bottom of this letter.

Please note that this journal operates with transparent peer review. This means that if your submission is accepted for publication, the full peer review history of your article will publish online alongside your article. This includes reviewer comments, editor decision letters, and your author responses.

The reviewers have provided useful, detailed feedback and suggest major revisions to your manuscript before we can consider it again for publication. In addition to reviewer feedback, we would welcome some discussion if and how one could incorporate wearable technology, recording devices, or augmented / virtual reality hardware into this field. I invite you to respond to the reviewers' comments and revise your manuscript within the next 60 days from today date.

To revise your manuscript, log into https://mc.manuscriptcentral.com/oxfnsc and enter your Author Centre, where you will find your manuscript title listed under "Manuscripts with Decisions." Under "Actions," click on "Create a Revision." Your manuscript number has been appended to denote a revision.

You may also click the below link to start the revision process (or continue the process if you have already started your revision) for your manuscript. If you use the below link you will not be required to login to ScholarOne Manuscripts.

*** PLEASE NOTE: This is a two-step process. After clicking on the link, you will be directed to a webpage to confirm. ***

https://mc.manuscriptcentral.com/oxfnsc?URL_MASK=32ef10db55e945c9bd77fdef0a83c6bd

You will be unable to make your revisions on the originally submitted version of the manuscript. Instead, revise your manuscript using a word processing program and save it on your computer. Please also highlight the changes to your manuscript within the document by using the track changes mode in MS Word or by using bold or colored text.

Once the revised manuscript is prepared, you can upload it and submit it through your Author Centre.

When submitting your revised manuscript, you will be able to respond to the comments made by the reviewer(s) in the space provided. You can use this space to document any changes you make to the original manuscript. In order to expedite the processing of the revised manuscript, please be as specific as possible in your response to the reviewer(s).

IMPORTANT: Your original files are available to you when you upload your revised manuscript. Please delete any redundant files before completing the submission.

Because we are trying to facilitate timely publication of manuscripts submitted to the Oxford Open Neuroscience, your revised manuscript should be uploaded within the next 60 days from today date. If it is not possible for you to submit your revision in a reasonable amount of time, we may have to consider your paper as a new submission.

Once again, thank you for submitting your manuscript to the Oxford Open Neuroscience and I look forward to receiving your revision.

Sincerely,

Prof. Alicia Izquierdo

Senior Editor, Oxford Open Neuroscience

aizquie@psych.ucla.edu

Reviewer: 1

Comments to the Author

Dear author(s),

Thank you for your hard work on this manuscript. Overall, I think you have justified why neuroscientific methods can be used to study theatre, but I think more evidence and explanation is required to justify what theatre neuroscience can teach us about human neuroscience more broadly, especially as you’ve tried to make a case for greater funding for this type of research. Reading of the article would be enhanced if the writing style was more scholarly and referencing errors corrected.

Please see specific comments below:

Writing style:

- Page 1 lines 17-22 “social cognitive neuroscientists have shifted their focus towards two-person and real-world neuroscience, where the former involves measuring social behaviour that is not isolated from another person or the environment” – I think this is unusual phrasing and oversells how naturalistic current neuroimaging technology is.

- Page 6, lines 13-14 “ and that is okay” doesn’t sound very scholarly.

- I think the ideas in the first 3 paragraphs on page 1 could be more organised. I feel like you jumped around a bit when discussing hyperscanning and other neurosciences techniques.

- I’m not sure about the uses of “I” through-out. Is there only one author? The uses of “we” and “our” later implies there’s multiple.

- The proposed definition of theatre neuroscience seems a bit long and imprecise in my personal opinion.

- Unfortunately, the line numbers don’t match the lines of text, which make referencing specific lines slightly imprecise.

- Several instances of incorrect in-text references e.g., page 3 line 36 and page 4 line 12.

Content:

- The author(s) mentioned the neuroscience of the audience is relevant to theatre neuroscience e.g., page 2 line 22-23. The rest of the article focuses on the actors, with minimal mention of the audience. To an outsider, the audience seems like a relatively big part of theatre.

- I’m not sure the “Why theatre” section fully explains why theatre neuroscience is more ecologically valid than laboratory neuroscience, except in specific theatre contexts. Specifically, on page 2 line 54-56 you mention that it’s more practical to use theatre to study walk through a city at night, but I don’t understand why is creating a theatre setup is more practical. Creating good staging and finding enough space to do this seems just as impractical as filling out the paper work to do this in a naturalistic setting?

- Is there any research to support the author(s) notion that theatre research is more ecologically valid than the lab?

- Beginning page 3, I wonder why are there more options for theatre staging than in the lab? If immersion is the main goal, then there’s no need to put the props on a stage, we could just put the props in the lab?

- Paragraphs 8-11 don’t contain any references and so don’t provide any evidence to support authors opinions.

- Paragraph 2 page 4 was the best evidence to support what theatre neuroscience brings to the neuroscience field, but the author(s) need to go a bit further to explain the utility and reference studies with convergent findings even if they are outside of the neuroscience field. Sense of self might be a good example of a topic that theatre neuroscience can offer a unique perspective on, but author needs to explain this to reader.

- Author highlights the greater expenses that are incurred during theatre neuroscience, but not why this is worthwhile investment, which is crucial give the restrictions on research funding in the current climate.

Miscellaneous:

- I’m slightly confused by the mention of the authors race and nationality. I understand that in qualitative psychology an understanding of the researchers inherent bias is important, but I’m slightly confused of the relevance in this more quantitative context. Additionally, there’s also no explanation of how the authors background effects their opinion on the topic. It’s possible I’ve missed the point of this statement in which case further explanation might be required.

Reviewer: 2

Comments to the Author

This Brief Perspective introduces Theatre-Neuroscience as an emerging interdisciplinary framework that integrates theatrical practice with social cognitive neuroscience to study social cognition in ecologically valid yet controlled settings. Drawing on neuroaesthetics, portable neuroimaging, and the author’s own empirical work, the paper argues that theatre offers a unique middle ground between laboratory experiments and uncontrolled real-world scenarios, with particular relevance for studying self–other processing, interpersonal coordination, and brain–brain synchrony. The perspective outlines the conceptual motivations, reviews early empirical examples, discusses challenges of interdisciplinary collaboration, and highlights broader implications for neuroscience, the performing arts, and education.

The core idea is compelling and timely, and the motivation—especially the connection between theatre, neuroaesthetics, and social neuroscience—is genuinely interesting. I particularly appreciated how the author(s) frame theatre as a structured, repeatable, and socially rich environment that sits between traditional lab paradigms and fully naturalistic settings. However, even for a perspective piece, the current manuscript feels too superficial, especially on the neuroscience side, and would benefit from clearer structure, greater conceptual depth, and more precise scholarly framing.

Major comments

1. Tone and academic style: The manuscript would benefit from a more consistently formal academic tone. While it is appropriate for a perspective to include the author(s)’ own experiences and positionality, some sections read as overly colloquial. Tightening the language and reducing informal phrasing would strengthen the paper’s credibility and clarity, especially for a neuroscience audience.

2. Citation and referencing style: The way prior work is cited should be standardized throughout. For example, references should follow conventional formats such as Brown et al. (2019) when integrated into the sentence, or (Brown et al., 2019) when used parenthetically. Currently, inconsistent formatting (including extra parentheses) interrupts readability and should be corrected across the manuscript.

3. Clearer articulation of why theatre-based experiments are uniquely valuable: The authors touch on this point, but it could be made much clearer and more concise. In particular: (1) What specific advantages do theatre-based paradigms offer over traditional lab experiments? (2) Why are they preferable, in some cases, to fully real-world neuroscience studies? A short, well-structured synthesis explicitly comparing lab-based, theatre-based, and real-world approaches would substantially strengthen the argument.

4. Systematic discussion of neuroscience questions suited to theatre-neuroscience: The paper mentions several examples (e.g., self–other processing, interpersonal coordination, brain–brain synchrony), but these are presented somewhat descriptively. I encourage the authors to organize this section more systematically—for instance, by outlining categories of neuroscientific questions that theatre-neuroscience is particularly well-suited to address, and explaining why theatre is advantageous for each.

5. Participant-specific perspectives (actors, audiences, others): The distinction between different participant roles in theatre (e.g., actors, audiences, and potentially directors or ensembles) is an important strength of the proposed framework. However, this idea remains underdeveloped in the current manuscript. The paper would benefit from a clearer articulation of the neuroscience implications from each participant’s perspective—for example, how neural processes might differ between performing, observing, and co-present individuals. Even at the level of a perspective, outlining a small number of role-specific hypotheses (e.g., differential engagement of self-referential processing in actors versus predictive or mirroring processes in audiences) would add conceptual depth and provide more concrete guidance for future empirical work.

6. Challenges from a scientific rigor perspective: The challenges section focuses primarily on funding and collaboration, which are important, but it would benefit from a more explicit discussion of scientific and methodological concerns. Many neuroscientists remain skeptical of naturalistic paradigms due to issues of experimental control, reproducibility, and interpretability. Addressing these concerns directly—such as how theatre-based paradigms can mitigate, rather than exacerbate, these issues—would make the paper more balanced and persuasive.

Associate Editor

Comments to the Author:

Thank you for your submission. Theatre-neuroscience is an exciting emerging topic and would be of genuine interest to the readers. Overall, reviewers felt that the manuscript required major revisions that broadly include improving the systemic discussion and justification of theatre neuroscience and, revisions of the writing style and tone to reflect a scholarly and formal tone.

**Author's Response to Decision Letter for (OXFNSC-2026-001)**

Dear Associate Editor and Reviewers,

Thank you for taking the time to read and review my article. Your suggestions have been greatly received, as I agree that my article needed strengthening and correcting in the areas highlighted. The article has been restructured based on comments 18-20 to present the research that has occurred in Theatre-Neuroscience so far on audiences and actors in the areas of interpersonal synchrony and an actor’s sense of self, as this has been the predominant focus of the field. I believed this was the right direction to go, as the field is emerging, so the empirical support for arguments, especially regarding ecological validity, are in progress. The evaluation of the field remains as it was in the first version of this article, with relevant corrections. The abstract has also been edited to align with the direction of the article.

Below are my responses to your comments; text in “speech marks” are extracts of the revised text from the article.


Reviewer: 1

Writing style:

1. Page 1 lines 17-22 “social cognitive neuroscientists have shifted their focus towards two-person and real-world neuroscience, where the former involves measuring social behaviour that is not isolated from another person or the environment” – I think this is unusual phrasing and oversells how naturalistic current neuroimaging technology is.

Response: This paragraph has been removed as it is no longer needed.

2. Page 6, lines 13-14 “ and that is okay” doesn’t sound very scholarly.

Response: This statement has been removed.

3. I think the ideas in the first 3 paragraphs on page 1 could be more organised. I feel like you jumped around a bit when discussing hyperscanning and other neurosciences techniques.

Response: The first three paragraphs have been restructured to suit the new direction of the article and no longer discuss hyperscanning and other neuroimaging techniques in this way.

4. I’m not sure about the uses of “I” through-out. Is there only one author? The uses of “we” and “our” later implies there’s multiple.

Response: This was my first attempt at writing a solo article; I’m normally accustomed to writing papers with many authors, hence slipped into the collective pronoun. Here, I clarify that I am the only author, with my supervisor as my editor, whom I mentioned in my Acknowledgements.

5. The proposed definition of theatre neuroscience seems a bit long and imprecise in my personal opinion.

Response: I have simplified the definition of Theatre-Neuroscience, still aligning it to what is true of my practice and the research that has been conducted so far in the field. As there is no official definition and my previous paper (Greaves et al., 2022) was the first mention of the term, I hope to have some autonomy in providing my definition of what the field is.

“Theatre-Neuroscience is an emerging interdisciplinary field of research that interpolates neuroscientific techniques into the investigation of the creation and experience of dramatic acting, and the effects of acting interventions on cognition and behaviour (as presented in Greaves et al., (2022)).” Pg. 1 Paragraph 1

6. Unfortunately, the line numbers don’t match the lines of text, which make referencing specific lines slightly imprecise.

Response: I may have a misunderstanding of this comment but if it is towards formatting, I did not add specific formatting to the text as the ‘Preparing your manuscript’ guidelines do not provide any, hence why the line numbers may not match. I have now justified the text with 1.5 spacing between lines for clarity of reading.

7. Several instances of incorrect in-text references e.g., page 3 line 36 and page 4 line 12.

Response: This was an error in not overriding my referencing software. I assumed these edits would be corrected when the journal restructures the article to its designated format. I have now made these corrections.

Content

8. The author(s) mentioned the neuroscience of the audience is relevant to theatre neuroscience e.g., page 2 line 22-23. The rest of the article focuses on the actors, with minimal mention of the audience. To an outsider, the audience seems like a relatively big part of theatre.

Response: I have now included a section for research on audiences. This research does not use neuroimaging techniques, but I thought it was useful to add, as it focuses on social cognition, was conducted by neuroscientific research teams, and can be used as inspiration for future neuroimaging studies.

9. I’m not sure the “Why theatre” section fully explains why theatre neuroscience is more ecologically valid than laboratory neuroscience, except in specific theatre contexts. Specifically, on page 2 line 54-56 you mention that it’s more practical to use theatre to study walk through a city at night, but I don’t understand why is creating a theatre setup is more practical. Creating good staging and finding enough space to do this seems just as impractical as filling out the paper work to do this in a naturalistic setting?

Response: This section is no longer included.

10. Is there any research to support the author(s) notion that theatre research is more ecologically valid than the lab?

Response: This argument is no longer included; instead, my mentions of ecological validity address the idea that the lab can produce ecologically valid stimuli or the lab can be brought to ecologically valid environments.

“…providing examples of how neuroscientists can improve the ecological validity (Schmuckler, 2001) of stimuli or research environments by either integrating performing arts into lab-based stimuli or integrating the lab into performing arts environments (Calvo-Merino et al., 2005; von Zimmermann et al., 2018).” Page 1-2 Paragraph 2

11. Beginning page 3, I wonder why are there more options for theatre staging than in the lab? If immersion is the main goal, then there’s no need to put the props on a stage, we could just put the props in the lab?

Response: This argument is no longer included.

12. Paragraphs 8-11 don’t contain any references and so don’t provide any evidence to support authors opinions.

Response: The ‘Why Theatre’ section that contained these paragraphs has been removed and replaced with empirical evidence of what the field has researched so far.

13. Paragraph 2 page 4 was the best evidence to support what theatre neuroscience brings to the neuroscience field, but the author(s) need to go a bit further to explain the utility and reference studies with convergent findings even if they are outside of the neuroscience field. Sense of self might be a good example of a topic that theatre neuroscience can offer a unique perspective on, but author needs to explain this to reader.

Response: The article has now been restructured to discuss audience and actor research, with audience research focusing on physiological and behavioural measures, and actor research focusing on neuroimaging measures. I hope this shed more light and empirical support for questions Theatre-Neuroscience can answer in topics of interpersonal synchrony and an actor’s sense of self.

14. Author highlights the greater expenses that are incurred during theatre neuroscience, but not why this is worthwhile investment, which is crucial give the restrictions on research funding in the current climate.

Response: I have now included better justifications as to why the field should be funded i.e.,

“However, publications such as Goldstein & Winner (2024)’s ‘Why Theatre Education Matters’ are influential in deconstructing and explaining the benefits of theatre training for the strengthening of social cognitive skills such as theory of mind, empathy and emotional regulation. Awaiting empirical support creates a stagnant loop as funding needs to be secured to conduct research, but the research is initially needed to provide grounds for funding.” Pg.6 Paragraph 2

Miscellaneous

15. I’m slightly confused by the mention of the authors race and nationality. I understand that in qualitative psychology an understanding of the researchers inherent bias is important, but I’m slightly confused of the relevance in this more quantitative context. Additionally, there’s also no explanation of how the authors background effects their opinion on the topic. It’s possible I’ve missed the point of this statement in which case further explanation might be required.

Response: I initially misunderstood whether there needed to be an explicit relation of my work to the Black community, as it is not direct in my research topics. Now I know this isn’t necessary, so I have removed this statement.

Reviewer: 2

16. Tone and academic style: The manuscript would benefit from a more consistently formal academic tone. While it is appropriate for a perspective to include the author(s)’ own experiences and positionality, some sections read as overly colloquial. Tightening the language and reducing informal phrasing would strengthen the paper’s credibility and clarity, especially for a neuroscience audience.

Response: I believe I have corrected my academic tone and style of writing. I hope this reads more aligned with the tone of what is expected from a Neuroscientific brief perspective.

17. Citation and referencing style: The way prior work is cited should be standardized throughout. For example, references should follow conventional formats such as Brown et al. (2019) when integrated into the sentence, or (Brown et al., 2019) when used parenthetically. Currently, inconsistent formatting (including extra parentheses) interrupts readability and should be corrected across the manuscript.

Response: This was an error in not overriding my referencing software. I assumed these edits would be corrected when the journal restructures the article to its format. I have now made these corrections.

18. Clearer articulation of why theatre-based experiments are uniquely valuable: The authors touch on this point, but it could be made much clearer and more concise. In particular: (1) What specific advantages do theatre-based paradigms offer over traditional lab experiments? (2) Why are they preferable, in some cases, to fully real-world neuroscience studies? A short, well-structured synthesis explicitly comparing lab-based, theatre-based, and real-world approaches would substantially strengthen the argument.

19. Systematic discussion of neuroscience questions suited to theatre-neuroscience: The paper mentions several examples (e.g., self–other processing, interpersonal coordination, brain–brain synchrony), but these are presented somewhat descriptively. I encourage the authors to organize this section more systematically—for instance, by outlining categories of neuroscientific questions that theatre-neuroscience is particularly well-suited to address, and explaining why theatre is advantageous for each.

20. Participant-specific perspectives (actors, audiences, others): The distinction between different participant roles in theatre (e.g., actors, audiences, and potentially directors or ensembles) is an important strength of the proposed framework. However, this idea remains underdeveloped in the current manuscript. The paper would benefit from a clearer articulation of the neuroscience implications from each participant’s perspective—for example, how neural processes might differ between performing, observing, and co-present individuals. Even at the level of a perspective, outlining a small number of role-specific hypotheses (e.g., differential engagement of self-referential processing in actors versus predictive or mirroring processes in audiences) would add conceptual depth and provide more concrete guidance for future empirical work.

Response: This is a response to comments 18-20. I have restructured the article using these comments as a direction. Rather than compare in-lab and real-world experiments, this article now presents and evaluates the research that has occurred so far in Theatre-Neuroscience in audiences and actors. I specifically focus on research on interpersonal synchrony and the sense of self, as these are the predominant areas of focus across these small number of publications.

21. Challenges from a scientific rigor perspective: The challenges section focuses primarily on funding and collaboration, which are important, but it would benefit from a more explicit discussion of scientific and methodological concerns. Many neuroscientists remain skeptical of naturalistic paradigms due to issues of experimental control, reproducibility, and interpretability. Addressing these concerns directly—such as how theatre-based paradigms can mitigate, rather than exacerbate, these issues—would make the paper more balanced and persuasive.

Response: Alongside the existing challenges mentioned, I have included a challenge regarding scientific rigour, i.e.,

“An initial challenge is that, as the field is newly emerging, consistent empirical support for its benefits are in development. The current and upcoming significant findings would need to be replicated for strong reliability and credibility.” Pg. 6 Paragraph 2

**Decision Letter (OXFNSC-2026-001.R1)**

**16-May-2026**

Dear Ms. Greaves,

Manuscript ID OXFNSC-2026-001.R1 entitled "Theatre-Neuroscience: building interdisciplinary paradigms to investigate social cognition" which you submitted to the Oxford Open Neuroscience, has been reviewed.  The comments of the reviewers are included at the bottom of this letter.

Please note that this journal operates with transparent peer review. This means that if your submission is accepted for publication, the full peer review history of your article will publish online alongside your article. This includes reviewer comments, editor decision letters, and your author responses.

One of the reviewers has recommended publication, but the other reviewer suggests additional revisions to your manuscript. Therefore, I invite you to respond to those remaining comments and revise your manuscript within the next 30 days from todays date. The Associate Editor also makes constructive suggestions to further improve the manuscript.

To revise your manuscript, log into https://mc.manuscriptcentral.com/oxfnsc and enter your Author Centre, where you will find your manuscript title listed under "Manuscripts with Decisions."  Under "Actions," click on "Create a Revision."  Your manuscript number has been appended to denote a revision.

You may also click the below link to start the revision process (or continue the process if you have already started your revision) for your manuscript. If you use the below link you will not be required to login to ScholarOne Manuscripts.

*** PLEASE NOTE: This is a two-step process. After clicking on the link, you will be directed to a webpage to confirm. ***

https://mc.manuscriptcentral.com/oxfnsc?URL_MASK=e6223af1e35b41aba409d7fe45981b78

You will be unable to make your revisions on the originally submitted version of the manuscript.  Instead, revise your manuscript using a word processing program and save it on your computer.  Please also highlight the changes to your manuscript within the document by using the track changes mode in MS Word or by using bold or colored text.

<strong>REMINDER:</strong> if you have not already done so, when submitting your revised manuscript, please include a short alternative-text description under the figure legend for each figure. Alt-text is required to ensure the accessibility of our publications. Guidance is available on the journal’s Author Guidelines webpage and at <a href="https://academic.oup.com/pages/for-authors/journals/preparing-and-submitting-your-manuscript" target="_new">https://academic.oup.com/pages/for-authors/journals/preparing-and-submitting-your-manuscript</a>

Once the revised manuscript is prepared, you can upload it and submit it through your Author Centre.

When submitting your revised manuscript, you will be able to respond to the comments made by the reviewer(s) in the space provided.  You can use this space to document any changes you make to the original manuscript.  In order to expedite the processing of the revised manuscript, please be as specific as possible in your response to the reviewer(s).

IMPORTANT:  Your original files are available to you when you upload your revised manuscript.  Please delete any redundant files before completing the submission.

Because we are trying to facilitate timely publication of manuscripts submitted to the Oxford Open Neuroscience, your revised manuscript should be uploaded within the next 30 days from todays date.  If it is not possible for you to submit your revision in a reasonable amount of time, we may have to consider your paper as a new submission.

Once again, thank you for submitting your manuscript to the Oxford Open Neuroscience and I look forward to receiving your revision.

Sincerely,
Prof. Alicia Izquierdo
Senior Editor, Oxford Open Neuroscience
aizquie@psych.ucla.edu


Reviewer: 2

Comments to the Author
The updated manuscript is much better organized, especially the author now focused on the audience and the actor side separately, and added some more in depth scientific discussion of the field.

Reviewer: 1

Comments to the Author
Thank you to the author for their further work on the manuscript.

Whilst I think the manuscript is massively improved, especially in terms of scholarly tone and writing style, I still find many of the arguments underdeveloped and unconvincing. The title suggests that the article will explain what theatre neuroscience can tell us about wider social cognition. Currently, I think the arguments are too specific to theatre neuroscience to convince the general reader than theatre neuroscience is something that should be prioritised in terms of funding and policy as suggested by the author.

I liked the section on how theatre educational interventions can benefit children with developmental disorders and I think this could be extended. A brief explanation of the proposed mechanism of how this intervention can help specific populations would be interesting. Secondly, towards the end it says “benefits of theatre training for the strengthening of social cognitive skills such as theory of mind, empathy and emotional regulation”. I think a more detailed explanation of these cognitive benefits would be more convincing.

I think the mention of synchrony between the audience is interesting, but I think it needs to more clearly explained how theatre neuroscience contributes the synchrony literature more broadly, additionally more tangible real world implications would highly strengthen the argument. For example, what would the benefits or implications of social connectedness between audience members be? Perhaps the author could consider if the presented findings might distinguish between competing theories of where synchrony comes from. One advantage of theatre neuroscience could be studying synchrony or just group dynamics between a large group of people. I can’t imagine a lab study where you would bring together such a large group.  A common criticism of hyperscanning is that participants are mostly pairs or small groups.
Unfortunately, I am still not convinced that theatre can make research more ecologically valid outside of research of actors or the audience. Much theatre staging is more visually appealing, more abstract or has different aspect ratios to the real world. Perhaps the author can explain in more detail or, given the short length of the piece, use the words to explain another point in more detail.

I find some of the author’s statements quite bold given the evidence provided was largely very specific to the theatre context:
- “For neuroscience, the field provides blueprints of how interdisciplinary collaborations can propel the development of knowledge about human cognition and behaviour” - If you could describe how researchers could piggy-back onto an existing theatre production to save money, I’d be slightly more convinced.
- “For policy, empirical findings that promote the benefits of applied acting interventions for social cognitive development can be used to support the funding of the educational sectors of the acting industry. This would demonstrate how neuroscience can be used to advocate for fields facing reduced financial support, e.g., as seen in research on the benefits of funding dance interventions for neurodegenerative conditions (Sharp & Hewitt, 2014)” need to be able to demonstrate more benefits than social connectedness between audience members for this to be true.
- “empirical support for the benefits of being an audience member” – same as above.

Some other miscellaneous comments:
- First line of intro, not sure interpolates is the correct word choice here
- Still reference formatting issues:
o “(as presented in Greaves et al., (2022))” should be “(as presented in Greaves et al., 2022)”.
o (i.e., visual arts) (Goldstein, 2009), should be (i.e., visual arts; Goldstein, 2009)

Associate Editor
Comments to the Author:
Dear authors,
Thank you for the adapted manuscript. I agree with reviewer that the text has significantly improved in scholarly tone and writing style, the context of the paper is incredibly interesting and provides added value to the field of social neuroscience. However, I do agree that the title does not reflect the text. Please consider a more general title along the lines of: “Theatre-Neuroscience: A brief perspective on the use of live performance to the study of social cognition.”

Regarding reviewer’s comment, please revise the following:
Expand the section to briefly discuss the cognitive benefits on children with developmental disorders. Similarly, expand on the synchrony section to include benefits of social connectedness and discuss competing theories. Lastly, revise sections with the noted bold statements.

Miscellaneous comments:
Pg12, 43-46: Is this true? Or is there a need to include theatre-neuroscience in broader discussions and groups who are also analysing complex datasets (potentially clinicians who has noisy data, navigation researchers, developmental researchers etc.)
Pg13, 55: This is a very interesting study and it would be nice to include a few discussion sentences/ implications here
Pg 14, 53: any examples?
Pg15, 19-28: I had expected some policy advocacy around the benefits of in-person, real world social interaction over digital here.

**Author's Response to Decision Letter for (OXFNSC-2026-001.R1)**

Dear Editor and Reviewer,
Thank you for further comments towards the improvement of my brief perspective. These comments have enabled me to reevaluate the main message I aim to bring forward. As a result, I have restructured the piece to focus on interpersonal coordination and the sense of self, as these are the two main areas of research in Theatre-Neuroscience. My intention for submitting this piece for the ‘Picture a Black in Neuro Scientist’ special edition was to present the research that I’ve been at the forefront of during my research career. I currently specialise in the actors’ sense of self, which may be why other interesting areas such as theatre and neurodiversity are not developed as much, because it is not a picture of the neuroscience that I am practising (maybe I’m taking the title of the special edition too literally). Nevertheless, the aim of this piece is for me to introduce Theatre-Neuroscience to the journal’s readers so that they are aware of newer neuroscientific creative disciplines. I also hope to ensure that my work is featured in this historical special edition, for documentation purposes. I think it’s important that unconventional research is included, as there are upcoming neuroscientists who are interested in interdisciplinary neuroscience and look forward to more publications supporting these intersections. Therefore, my case is no longer about funding; I understand how my ideas may need more development, and it’s a hard case to argue as the field is very new, so my main aim is to simply present the field, discuss what it has done and point towards future research. I hope an explanation of my motivations will aid the continual guidance of shaping this piece to meet journal expectations but also remain true to my research focus.

REVIEWER: 1

1. The title suggests that the article will explain what theatre neuroscience can tell us about wider social cognition. Currently, I think the arguments are too specific to theatre neuroscience to convince the general reader than theatre neuroscience is something that should be prioritised in terms of funding and policy as suggested by the author.

Response: The title has now been changed to ‘Theatre-Neuroscience: a brief perspective on the use of neuroscientific techniques to measure interpersonal coordination and the sense of self in theatrical contexts’

2. I liked the section on how theatre educational interventions can benefit children with developmental disorders and I think this could be extended. A brief explanation of the proposed mechanism of how this intervention can help specific populations would be interesting.

Response: I have now expanded on the Ward et al., (2019) study with its implications.

3. Secondly, towards the end it says “benefits of theatre training for the strengthening of social cognitive skills such as theory of mind, empathy and emotional regulation”. I think a more detailed explanation of these cognitive benefits would be more convincing.

Response: I did not expand on these studies as I aimed to focus on experiments which used neuroscientific techniques rather than self-reports. However, I mentioned them because paired with neuroscientific evidence they point towards the argument that applied theatre is beneficial for children and adolescents.

4. I think the mention of synchrony between the audience is interesting, but I think it needs to more clearly explained how theatre neuroscience contributes the synchrony literature more broadly, additionally more tangible real world implications would highly strengthen the argument. For example, what would the benefits or implications of social connectedness between audience members be? Perhaps the author could consider if the presented findings might distinguish between competing theories of where synchrony comes from.

Response: I have now expanded upon the importance of synchrony research in the ‘Interpersonal Coordination…’ and ‘Evaluating Theatre-Neuroscience’ sections.

5. Unfortunately, I am still not convinced that theatre can make research more ecologically valid outside of research of actors or the audience. Much theatre staging is more visually appealing, more abstract or has different aspect ratios to the real world. Perhaps the author can explain in more detail or, given the short length of the piece, use the words to explain another point in more detail.

Response: I have now shifted my focus from the ecological validity arguments; perhaps I will revisit this in a future article once more research develops in the field

6. I find some of the author’s statements quite bold given the evidence provided was largely very specific to the theatre context:
- “For neuroscience, the field provides blueprints of how interdisciplinary collaborations can propel the development of knowledge about human cognition and behaviour” - If you could describe how researchers could piggy-back onto an existing theatre production to save money, I’d be slightly more convinced.
- “For policy, empirical findings that promote the benefits of applied acting interventions for social cognitive development can be used to support the funding of the educational sectors of the acting industry. This would demonstrate how neuroscience can be used to advocate for fields facing reduced financial support, e.g., as seen in research on the benefits of funding dance interventions for neurodegenerative conditions (Sharp & Hewitt, 2014)” need to be able to demonstrate more benefits than social connectedness between audience members for this to be true.
- “empirical support for the benefits of being an audience member” – same as above.

Response: I have now rephrased my bold statements and reframed my evaluations section

SOME OTHER MISCELLANEOUS COMMENTS:

7. First line of intro, not sure interpolates is the correct word choice here

Response: I have now changed this term to ‘integration’

8. Still reference formatting issues:

o “(as presented in Greaves et al., (2022))” should be “(as presented in Greaves et al., 2022)”.
o (i.e., visual arts) (Goldstein, 2009), should be (i.e., visual arts; Goldstein, 2009)

Response: I have now fixed these formatting issues

ASSOCIATE EDITOR

9. I do agree that the title does not reflect the text. Please consider a more general title along the lines of: “Theatre-Neuroscience: A brief perspective on the use of live performance to the study of social cognition.”

Response: The title has now been changed to ‘Theatre-Neuroscience: a brief perspective on the use of neuroscientific techniques to measure interpersonal coordination and the sense of self in theatrical contexts’

Regarding reviewer’s comment, please revise the following:
Expand the section to briefly discuss the cognitive benefits on children with developmental disorders. Similarly, expand on the synchrony section to include benefits of social connectedness and discuss competing theories. Lastly, revise sections with the noted bold statements.

Response: I have now expanded upon the importance of synchrony research in the ‘Interpersonal Coordination…’ and ‘Evaluating Theatre-Neuroscience’ sections. I have slightly expanded on neurodiversity, but as explained in the opening of the letter, it is not my area of expertise, so I didn’t want to delve into this area too much as it doesn’t represent the neuroscience I currently specialise in.

MISCELLANEOUS COMMENTS:
Pg12, 43-46: Is this true? Or is there a need to include theatre-neuroscience in broader discussions and groups who are also analysing complex datasets (potentially clinicians who has noisy data, navigation researchers, developmental researchers etc.)

Response: I have rephrased to ‘These studies present the scope of live theatre research and contribute to the development of protocols and pipelines necessary for collecting and analysing the complex datasets that are derived from real-world environments.’ I have also moved this to the Evaluation section. Pg. 06

Pg13, 55: This is a very interesting study and it would be nice to include a few discussion sentences/ implications here

Response: I have expanded a bit more as I have reframed my evaluation section to further the discussion on the implications of this research.

Pg 14, 53: any examples?

Response: This statement has now been removed as this section is now reframed.

Pg15, 19-28: I had expected some policy advocacy around the benefits of in-person, real world social interaction over digital here.

Response: I hatve now included the statement ‘This research also promotes the importance of live theatre despite rapid developments in streaming platforms and remote entertainment (Ginman, 2013; Richardson, 2015; Simonot-Bérenger et al., 2025).’ Pg. 05-06

**Decision Letter (OXFNSC-2026-001.R2)**

**13-Aug-2026**

Dear Ms. Greaves,

It is a pleasure to accept your revised manuscript entitled "Theatre-Neuroscience: a brief perspective on the use of neuroscientific techniques to measure interpersonal coordination and the sense of self in theatrical contexts" in its current form for publication in the Oxford Open Neuroscience. 

Please note that this journal operates with transparent peer review. This means that  the full peer review history of your article will publish online alongside your article. This includes reviewer comments, editor decision letters, and your author responses.

Next steps
You will receive an email from no-reply@scipris.com within roughly one week. This is your invitation to sign up for an account with SciPris, Oxford University Press’ author portal hosted by Aptara. You will need to create an account if you do not already hold one. Please register or log into your account and follow the online instructions which will guide you through signing your licence and paying the APC. The email and the portal have clearly signposted support options if you need any help during this process.

Please note that SciPris is a completely different system from ScholarOne, so your credentials to submit your manuscript here will not work there. Once you’ve created a SciPris account, you will be able to use it whenever you publish with Oxford Open Neuroscience or any OUP journal. Please note that OUP will only ever request payment for applicable fees be made via SciPris or to an OUP bank account. If you ever have concerns about the legitimacy of a request, please do not hesitate to contact a customer services agent via the SciPris portal or directly via oupsupport@scipris.com.

Thank you for your fine contribution.  On behalf of the Editors of the Oxford Open Neuroscience, we look forward to your continued contributions to the Journal.

Sincerely,
Prof. Alicia Izquierdo
Senior Editor, Oxford Open Neuroscience
aizquie@psych.ucla.edu
